# Prevalence of maxillary sinus septa and its impact on sinus augmentation procedures for implant placement

**DOI:** 10.6026/973206300211123

**Published:** 2025-05-31

**Authors:** Nitin Purohit, Chinmayee Dighraskar, Aditi Singh, Lubna Tabassum Siddiqui, Abdul Kalam Azad, Nirav G. Patel, Debasis Mishra, Abhigyan Manas

**Affiliations:** 1 Department of Dentistry, District Hospital, Rudraprayag, Uttarakhand, India; 2 Department of Pediatric and Preventive Dentistry, New Horizon Dental College and Research Institute, Bilaspur, Chhattisgarh, India; 3 Department of Oral and Maxillofacial Surgery, Goenka Research Institute of Dental Sciences, Gandhinagar, Gujarat, India; 4 Department of Periodontics, Swargiya Dadasaheb Kalmegh Smruti Dental College and Hospital, Wanadongri, Hingna, Nagpur, Maharashtra, India; 5 Department of Oral and Maxillofacial Surgery, College of Dentistry, Qassim University, Saudi Arabia; 6 Department of Oral & Maxillofacial Surgery, Goenka Research Institute of Dental Sciences, Gandhinagar, Gujarat, India; 7 Intern, Kalinga Institute of Dental Sciences, KIIT Deemed to be University, Patia, Bhubaneswar-751024, Odisha, India; 8 Department of Oral and Maxillofacial Surgery, Faculty of Dental Sciences, Uttar Pradesh University of Medical Sciences, Saifai, Etawah, India

**Keywords:** Schneiderian membrane perforation, sinus augmentation, prevalence, implant success, maxillary sinus septa

## Abstract

The prevalence, classification and clinical significance of sinus septa and their impact on surgical procedures is of interest. A cross-sectional study involving 30 patients assessed maxillary sinus septa using CBCT imaging. Septa were classified into primary and secondary types and surgical difficulty, membrane perforation and implant success were recorded. Sinus septa are prevalent and significantly affect the difficulty of sinus augmentation and the risk of membrane perforation. Preoperative CBCT evaluation and careful planning are critical for successful outcomes.

## Background:

The anatomical variations within the maxillary sinus, particularly the presence of sinus septa, present notable challenges during sinus augmentation procedures aimed at facilitating dental implant placement [1]. Maxillary sinus septa, which are thin bony partitions within the sinus cavity, can vary in their orientation, size and location, often complicating the surgical field and increasing the technical difficulty of membrane elevation [2]. Such morphological variations have been significantly correlated with higher rates of Schneiderian membrane perforation, postoperative complications and compromised implant stability [3]. Consequently, a comprehensive understanding of the prevalence, type and clinical implications of these septa is paramount for meticulous surgical planning and minimizing intraoperative risks [4]. Despite the critical role of maxillary sinus septa in influencing surgical outcomes, there remains considerable variability in the reported prevalence and morphological classifications across different populations. Therefore, it is of interest to evaluate the prevalence, classification and clinical significance of sinus septa and their impact on surgical procedures.

## Materials and Methods:

This original cross-sectional observational study was conducted on 30 maxillary sinus cases to evaluate the prevalence and clinical significance of sinus septa. Each case was categorized according to two recognized classifications of maxillary sinus septa: the Underwood classification (1910) [5], which primarily describes congenital (primary) septa formed during maxillary growth and the Cavalcanti *et al.* (2018) [6], which identifies secondary septa associated with post-extraction alveolar bone resorption and sinus pneumatization. High-resolution cone-beam computed tomography (CBCT) imaging was employed to accurately visualize and assess the septa's morphology, measure its height (in millimeters) and determine its anatomical location (anterior, middle, or posterior region) within the sinus cavity. For each case, the complexity of sinus augmentation was graded using a standardized difficulty scale ranging from 1 (very easy) to 5 (very difficult), based on intraoperative findings [7] (Table 1). Furthermore, the incidence of Schneiderian membrane perforation was meticulously recorded during sinus lift procedures. Implant placement success, defined by proper three-dimensional positioning without membrane compromise, was also documented. All imaging analyses were conducted independently by two calibrated examiners to minimize observational bias and discrepancies were resolved through consensus. Data were statistically analyzed to evaluate the correlation between the type of septa and surgical difficulty, perforation risk and implant outcome, with significance levels set at p < 0.05. This grading system is used to classify the difficulty of sinus membrane elevation during sinus lift surgeries-commonly performed in dental implant procedures in the posterior maxilla where bone height is insufficient.

## Results:

In this cross-sectional observational study, a total of 30 maxillary sinus cases from 30 patients were evaluated to assess the prevalence, classification and clinical impact of maxillary sinus septa on surgical procedures and implant outcomes. The participants ranged in age from 35 to 70 years, with a mean age of 52.4 ± 9.6 years. The sample comprised 18 males (60%) and 12 females (40%), reflecting a modest male predominance (Table 2). Upon evaluation using high-resolution CBCT imaging, septa were identified in 19 of the 30 sinuses, accounting for a prevalence rate of 63.3%, while the remaining 11 cases (36.7%) exhibited no septa (Table 3). When classified according to the two recognized systems, the results were as follows: In the present study, primary septa as classified by Underwood were observed in 10 cases (33.3%), while secondary septa, as per Cavalcanti *et al.* were found in 9 cases (30%). Primary septa are congenital and usually located near the roots of maxillary molars, whereas secondary septa are acquired, often resulting from irregular sinus pneumatization after tooth loss. Identifying these septa types is crucial for pre-surgical planning, as primary septa tend to be more predictable, whereas secondary septa can increase the complexity of sinus lift procedures. Notably, 11 patients (36.7%) had no septa (Table 4). Anatomically, septa were most commonly located in the middle region of the sinus (26.7%), followed by the anterior region (23.3%) and posterior region (13.3%). The mean height of the identified septa was 6.1 ± 2.3 mm, with a range from 3.2 mm to 10.5 mm. Surgical difficulty was graded using a scale from 1 (very easy) to 5 (very difficult), based on factors like access, membrane integrity and intraoperative complications. The presence of septa was associated with higher difficulty grades. Table 5 has shown summary of the grading results. A statistically significant correlation between the type of septa and the surgical difficulty grade was observed, with a p-value of 0.012, underscoring the clinical relevance of preoperative septa assessment. Table 6 demonstrates that the presence and type of sinus septa significantly influence the difficulty of sinus membrane elevation procedures. Cases with primary septa had the highest mean difficulty score (4.3 ± 0.6), followed by those with secondary septa (3.0 ± 0.7), while cases without septa had the lowest difficulty (1.2 ± 0.4). The differences were statistically significant, with p-values of 0.005 and 0.018, respectively, indicating a clear association between septa type and surgical complexity. This emphasizes the importance of identifying septa during preoperative planning to anticipate potential challenges. Intraoperative perforation of the Schneiderian membrane a well-recognized complication during sinus lift procedures-was observed in 6 cases (20%). Among these: (Table 7). The statistical analysis revealed a significant association between the presence of septa and the risk of membrane perforation, with a p-value of 0.046 and an odds ratio of 3.6. This suggests that patients with maxillary sinus septa are approximately 3.6 times more likely to experience membrane perforation during augmentation procedures. Implant placement success, defined by accurate three-dimensional positioning without compromising membrane integrity, was achieved in 26 out of 30 cases (86.7%). Interestingly, all 11 cases without septa resulted in successful implant placement (100%), compared to 15 out of 19 cases (78.9%) with septa. Despite the clinical trend suggesting that septa may pose challenges to implant success, no statistically significant difference was observed (p = 0.118) (Table 8). To ensure the reliability of CBCT analysis, two calibrated examiners independently assessed all imaging parameters. The inter-observer variability was minimal, with a Cohen's Kappa score of 0.84, indicating high agreement. The agreement in septa height measurements was also strong, with a mean deviation of 0.3 mm ± 0.2 mm, validating the consistency of radiographic evaluations.

## Clinical significance:

The results of this study underscore the critical role of maxillary sinus septa in influencing both the surgical complexity and outcomes of sinus augmentation procedures. The observed correlation between septa presence and increased difficulty, as well as a higher risk of membrane perforation, highlights the need for careful preoperative evaluation using CBCT imaging. Understanding the type, location and morphology of sinus septa allows for tailored surgical planning, minimizing complications and enhancing implant success. While implant success was not significantly impacted by septa presence, the trend suggests a need for heightened caution in cases with complex septal anatomy, particularly when multiple septa are present. This study also demonstrates the importance of a structured approach to grading surgical difficulty and monitoring intraoperative complications, providing valuable insights for clinicians to optimize their surgical strategies and improve patient outcomes.

## Discussion:

Maxillary sinus septa present a significant challenge during sinus augmentation procedures. The results of this study confirm the prevalence of septa in the maxillary sinus (63.3%), which is consistent with previous studies that also reported a high incidence of septa in this anatomical area (Underwood, 1910; Cavalcanti *et al.* 2018) [5, 6. Septa can vary in size, location and morphology and these factors have been shown to influence the complexity of surgical procedures. In this study, the presence of septa was associated with higher surgical difficulty, as demonstrated by a statistically significant correlation between septa type and surgical difficulty grade (p = 0.012) [7]. This finding aligns with previous literature, which highlighted that septa can complicate access to the sinus cavity, increase the risk of membrane perforation and make elevation of the sinus membrane more challenging (Alshamrani *et al.* 2023; Taschieri *et al.* 2012)[8, 9]. The classification of septa into primary and secondary types was integral in understanding their impact on surgical difficulty. Primary septa, which are congenital, were associated with higher difficulty grades than secondary septa, as reported by several authors (Cavalcanti *et al.* 2018) [6]. Secondary septa, often resulting from post-extraction alveolar bone resorption and sinus pneumatization, were also found to complicate the procedure but to a lesser extent compared to primary septa [10]. Furthermore, the anatomical location of the septa also plays a crucial role in determining surgical difficulty. Septa located in the posterior region were found to contribute to more difficult procedures, likely due to their proximity to vital structures such as the posterior superior alveolar artery and the infraorbitalnerv [11]. The incidence of Schneiderian membrane perforation was significantly higher in sinuses with septa (26.3%) compared to those without (9.1%). This finding is consistent with prior studies, which report that septa increase the risk of membrane perforation due to their rigid structure, which can hinder the elevation of the sinus membrane (Yang *et al.* 2024) [12]. The odds ratio of 3.6 suggests that the presence of septa makes perforation more likely, underscoring the importance of preoperative assessment and careful surgical planning to mitigate this risk. In terms of implant success, the study found no statistically significant difference between sinuses with and without septa (p = 0.118). While the trend suggested that sinuses without septa had a higher implant success rate (100%), this difference was not significant. This observation aligns with previous findings that, while septa may increase the complexity of sinus lift procedures, they do not necessarily impact the long-term success of implant placement when the procedure is carefully planned [13]. Abesi *et al.* (2023) analyzed the prevalence and anatomical characteristics of maxillary sinus septa, finding that septa were present in 34.7% of cases, with 22.9% having them unilaterally and 17.7% bilaterally. The prevalence was higher in men (51.9%) compared to women (47.0%). Septa were most commonly found in the middle sinus region (54.0%) and oriented coronal (56.9%). Additionally, 68.8% of septa were incomplete. The study underscores the significant prevalence and anatomical variability of maxillary sinus septa, emphasizing their potential clinical relevance in sinus-related procedures [14]. Henriques *et al.* (2023) conducted a meta-analysis of 35 studies (14,664 sinuses) and found an overall mean sinus septa prevalence of 33.2% per sinus (95% CI: 27.8-38.5%), and 41.0% per patient (95% CI: 36.0-46.0%) across 42 studies (9631 patients). The odds ratio (OR) for the difference in prevalence between sexes was 0.785 (P = 0.098), indicating no significant sex-based differences. Septa were commonly located in the middle area of the sinus and oriented transversely (86%). The study suggests that the presence of sinus septa, particularly in the middle region, can pose challenges during sinus floor elevation for implant rehabilitation [15]. Dragan *et al.* (2017) evaluated 200 maxillary sinus septa from 100 dentate and 100 edentate patients using three-dimensional cone-beam computed tomography (CBCT) reconstructions. They measured the length, lateral height, and thickness of the septa in the middle and medial regions, as well as the angle between the septum and the maxillary plane. The results revealed no significant difference between intraobserver measurements (p>0.05). However, middle height showed a significant difference between dentate and edentate patients (p=0.0095), with edentate patients having a lower mean. Most septa were located in the posterior region, and the majority of septa were oblique in orientation (81.2% in dentate patients and 53% in edentate patients). The study concluded that the maxillary sinus septum could potentially serve as an alternative site for implant placement, providing valuable insights for clinical procedures [16]. However, the reduced success rate in sinuses with septa (78.9%) emphasizes the need for careful planning, particularly in cases with multiple or posterior septa. The presence of maxillary sinus septa significantly influences the difficulty of sinus augmentation procedures. Septa are associated with higher surgical complexity, increased risk of Schneiderian membrane perforation and a lower implant success rate. However, when evaluated preoperatively using CBCT imaging and with careful surgical planning, these challenges can be effectively managed. Although septa do not significantly impact implant success in the long term, their presence highlights the need for meticulous surgical techniques to minimize complications and optimize outcomes.

## Conclusion:

Maxillary sinus septa are prevalent and significantly impact the surgical difficulty of sinus augmentation procedures, increasing the risk of Schneiderian membrane perforation. Careful preoperative evaluation and planning using CBCT imaging are essential for minimizing complications and ensuring optimal implant outcomes.

## Limitation of study:

A limitation of this study is the relatively small sample size, which may affect the generalizability of the results to larger populations.

## Future perspectives:

Future studies with larger sample sizes are needed to further investigate the relationship between sinus septa and implant success and to explore advanced surgical techniques for managing complex sinus anatomies.

## Figures and Tables

**Table 1:** Intraoperative difficulty grading criteria for sinus augmentation

**Grade**	**Label**	**Criteria**
1	Very easy	No septa; thick membrane; easy access; minimal elevation required
2	Easy	Thin membrane; moderate elevation; no septa
3	Moderate	Mild septa; minor complications; manageable elevation
4	Difficult	Primary/secondary septa present; risk of membrane perforation
5	Very difficult	Multiple septa; poor access; membrane perforation or intraoperative bleeding

**Table 2:** Demographic distribution

**Parameter**	**Value**
Age Range	35-70 years
Mean Age (± SD)	52.4 ± 9.6 years
Male Patients	18 (60%)
Female Patients	12 (40%)
This table provides demographic
data of a study population

**Table 3:** Prevalence of maxillary sinus septa

**Presence of Septa**	**Number of Cases**	**Percentage**
Yes	19	63.30%
No	11	36.70%
This table shows the
prevalence of sinus septa
(bony partitions inside the maxillary sinus)
in the studied population.

**Table 4:** Distribution of types of maxillary sinus septa

**Classification**	**Number of Cases**	**Percentage**
Underwood (Primary Septa)	10	33.30%
Cavalcanti et al. (Secondary Septa)	9	30%
This table presents a classification of
sinus septa types observed in the study population,
based on two commonly used anatomical classifications:
Underwood's (primary septa) and
Cavalcanti *et al.*'s (secondary septa).

**Table 5:** Surgical difficulty grading (intraoperative findings)

**Grade**	**No. of Cases**	**Associated with Septa**	**Percentage**
1 (Very Easy)	6	0	20%
2 (Easy)	7	1	23.30%
3 (Moderate)	5	3	16.70%
4 (Difficult)	7	6	23.30%
5 (Very Difficult)	5	5	16.70%
This table provides a detailed
analysis of the difficulty
grades of sinus membrane elevation
in relation to the presence of septa,
highlighting how anatomical
complexity impacts surgical challenges.

**Table 6:** Correlation between septa type and sinus augmentation difficulty (Overall ANOVA p = 0.012)

**Septa Type**	**Mean Difficulty Score (± SD)**	p-value vs. Absent*
Primary	4.3 ± 0.6	0.005*
Secondary	3.0 ± 0.7	0.018*
Absent (Ref.)	1.2 ± 0.4	-
*Compared to "Absent" group; p< 0.05
denotes statistical significance.
This table shows the relationship
between the type of sinus septa and the
difficulty score of sinus
membrane elevation procedures,
with statistical significance
indicated by p-values

**Table 7:** Incidence of schneiderian membrane perforation

**Septa Presence**	**Perforation Cases**	**Percentage**
With Septa	19-May	26.30%
Without Septa	11-Jan	9.10%
This table highlights the correlation between the presence
of sinus septa and the incidence of Schneiderian membrane
perforation during sinus elevation procedures.

**Table 8:** Implant success rate

**Septa Presence**	**Successful Implant Placement**	**Percentage**
With Septa	15/19	78.90%
Without Septa	11-Nov	100%
This table shows the relationship
between the presence of septa and the
success rate of implant placement
during sinus elevation procedures.
